# Comparison of Resting-State EEG Network Analyses With and Without Parallel MRI in Genetic Generalized Epilepsy

**DOI:** 10.1007/s10548-023-00977-6

**Published:** 2023-06-24

**Authors:** Daniel van de Velden, Christina Stier, Raviteja Kotikalapudi, Ev-Christin Heide, David Garnica-Agudelo, Niels K. Focke

**Affiliations:** 1 Clinic for Neurology, University Medical Center Göttingen, 37075 Göttingen, Germany; 2 Department of Neurology and Epileptology, Hertie Institute of Clinical Brain Research, University Medical Center Tübingen, University of Tübingen, 72076 Tübingen, Germany; 3 Clinic for Neurology, University Medical Center Essen/University Duisburg-Essen, 45147 Essen, Germany

**Keywords:** Hd-EEG, fMRI, Resting-state, Genetic generalized epilepsy, Brain network connectivity, Thalamus

## Abstract

Genetic generalized epilepsy (GGE) is conceptualized as a brain disorder involving distributed bilateral networks. To study these networks, simultaneous EEG-fMRI measurements can be used. However, inside-MRI EEG suffers from strong MR-related artifacts; it is not established whether EEG-based metrics in EEG-fMRI resting-state measurements are suitable for the analysis of group differences at source-level. We evaluated the impact of the inside-MR measurement condition on statistical group comparisons of EEG on source-level power and functional connectivity in patients with GGE versus healthy controls. We studied the cross-modal spatial relation of statistical group differences in seed-based FC derived from EEG and parallel fMRI. We found a significant increase in power and a frequency-specific change in functional connectivity for the inside MR-scanner compared to the outside MR-scanner condition. For power, we found reduced group difference between GGE and controls both in terms of statistical significance as well as effect size. Group differences for ImCoh remained similar both in terms of statistical significance as well as effect size. We found increased seed-based FC for GGE patients from the thalamus to the precuneus cortex region in fMRI, and in the theta band of simultaneous EEG. Our findings suggest that the analysis of EEG functional connectivity based on ImCoh is suitable for MR-EEG, and that relative group difference in a comparison of patients with GGE against controls are preserved. Spatial correspondence of seed-based FC group differences between the two modalities was found for the thalamus.

**Supplementary Information:**

The online version contains supplementary material available at 10.1007/s10548-023-00977-6.

## Introduction

Electroencephalography (EEG) and functional magnetic resonance imaging (fMRI) are typical modalities for imaging the human brain and its network functions. Both methods can be used either individually or combined as parallel EEG-fMRI and are of particular importance for the investigation of epilepsy and seizures. Approximately 50 million people worldwide suffer from epilepsy, and 15–30% of all diagnosed epilepsy cases are classified as genetic generalized epilepsy (GGE) (Beghi et al. 2019; Jallon and Latour 2005). GGE is regarded as a genetically caused epilepsy syndrome involving distributed bilateral networks. One approach to reaching a better and more detailed understanding of the pathophysiology in patients with GGE is the investigation of alterations in their brain networks with neuroimaging techniques.

There are two commonly used approaches in studying GGE cohorts: (i) analysis of task-based data, usually focusing on the occurrence of (generalized) spike wave discharges (GSWD) in epilepsy patients, (ii) and the analysis of resting-state.

In the first type of studies, EEG is used primarily to define the occurrence of GSWD, and their coupled perfusion changes appearing in fMRI are then analyzed. This approach allows one to assess the potential generators of GSWD, i.e. EEG patterns that are typical for generalized epilepsies in the interictal state. Multiple studies using EEG to define the timing of GSWD found a deactivation of the DMN regions in patients with GGE, with an activation in the thalamus (Aghakhani et al. 2004; Benuzzi et al. 2012; Gotman et al. 2005; Hamandi et al. 2006; Szaflarski et al. 2013). Those event-based studies focused on determining the development of the epileptic activity and the participating brain regions. Klamer et al. analyzed the BOLD time courses in patients with GGE in a time span before and after GSWD and found a similar deactivation of the DMN and activation of the thalamus region during the GSWD. They further applied dynamic causal modelling to calculate directed/effective connectivity results. They were able to show that two regions of the DMN, i.e. the precuneus cortex (PCC) and medial prefrontal cortex are key regions behind these connectivity changes (Klamer et al. 2018). Another study found a core network structure, consisting of the thalamus, and parietal cortex, among others in children with absence seizures that is involved in the GSWD activity (Carney et al. 2012, 2010; Masterton et al. 2012). A recent study provided evidence that there is a spatially specific power change and a change in FC between brain regions before GSWD (Tangwiriyasakul et al. 2018).

The other common approach is to study the functional brain state at rest, i.e. without external stimulus and (often) without knowledge of the occurrence of GSWD. Such studies allow insights into the default state of the brain since, in most patients, GSWD are relatively rare und brief events. Hence, functional connectivity (FC) analysis on resting-state data of patients with GGE allows to study persistent brain network alterations within the default mode and other networks. From such analyses, frontal and parietal brain regions were found to have a significant decrease in seed-based FC in patients with GGE.

McGill et al. found decreased seed-based FC from PCC and medial prefrontal cortex to the prefrontal region in patients with GGE (McGill et al. 2012). In another study, seed-based FC was applied on resting-state fMRI and showed a significant increase in FC in posterior DMN regions for the thalamus seed (Ji et al. 2015).

In contrast to fMRI, which typically probes vascular changes, EEG and/or MEG can investigate the neuronal signal directly. Similar to fMRI studies, early EEG/MEG studies focused on the analysis of the GSWD to localize the origin and characterize these GSWD (Holmes et al. 2004; Westmijse et al. 2009Miao et al. 2014).

Recently, increases in resting-state MEG and EEG source-level connectivity and power were found in patients with GGE compared to healthy controls (HC). FC was mainly increased in frontal, central, and inferior parietal regions of the brain, whereas power was increased in central, temporo-parietal regions and subcortical structures (Elshahabi et al. 2015; Li Hegner et al. 2018). In a further study, hd-EEG (high-density EEG) and MEG were used to analyze differences between patients with GGE and healthy controls and confirmed previously found regions with increased FC (Stier et al. 2021). Using hd-EEG, the largest FC increases were found in the theta frequency band in frontotemporal and central regions (Stier et al. 2022).

Currently, it is not established if EEG metrics based on inside MR-scanner EEG, such as power or functional connectivity, can be used for group differences at source-level. One limiting factor is the presence of strong artifacts contaminating the EEG measurements. At first, the rapid changes of the MR scanner gradients and the currents/potentials induced by them are by orders of magnitude stronger than the brain’s voltage fields and completely dominate the EEG signal (Hoffmann et al. 2000; Anami et al. 2003; Garreffa et al. 2003). Secondly, the ballistocardiogram (BCG), which is generated by pulse-related movement of the EEG electrodes and cables in the strong magnetic field in the MR scanner, is also a source of strong artifact in within scanner EEG (Allen et al. 1998). Different tools were developed to remove the gradient artifact (GA) and BCG artifacts from the EEG sensor data. A common approach is average artifact subtraction followed by removal of the residuals and BCG artifacts using an optimal basis set algorithm (OBS) (Niazy et al. 2005). The aim of correcting BCG artifacts led to several studies that improved our understanding of the origin and properties of BCGs and their influence on EEG data in parallel fMRI recordings (Debener et al. 2008; Mantini et al. 2007; Masterton et al. 2007; Mullinger et al. 2008; Vasios et al. 2006). Furthermore, it is known from the literature that beamformer source reconstruction methods are able to attenuate BCG artifacts in EEG-fMRI data (Brookes et al. 2008; Uji et al. 2021). A previous study on focal epilepsy patients showed that simultaneous electrical source imaging and fMRI analysis to be a robust approach of combining both modalities (Vulliemoz et al. 2009).

It is not established whether metrics such as power and FC at the source level of resting-state EEG in a parallel measurement of MRI are useful to detect known group differences in patients with GGE. Hence, one goal of this study was to clarify if simultaneous hd-EEG can still be used for source-reconstructed power and connectivity analysis for patients with GGE and if, despite the extensive data cleaning, the results are comparable to outside-MR scanner EEG. Therefore, we did an hd-EEG source-level connectivity and power analysis in patients with GGE versus healthy controls measured both inside (with parallel fMRI) as well as outside the MR scanner. We hypothesize that it is possible to reproduce the previously reported group difference of increased connectivity and power in patients with GGE observed in our previous works in the same cohort from EEG data measured with a parallel fMRI (Stier et al. 2021, 2022). In addition, we wanted to investigate whether seed-based multimodal connectivity analysis is suitable for simultaneous EEG and fMRI.

## Methods

### Participants

This study used the same subjects were used as in our previous work on outside-MR EEG (Stier et al. 2022), which included 28 patients diagnosed with GGE based on the International League Against Epilepsy classification (Scheffer et al. 2017). Furthermore, 50 healthy controls (HC) were recruited. HC had never experienced any seizures, were free of any neurological and psychiatric diseases, and were not taking any medication. Of these, a total of 15 patients with GGE and 16 HC had simultaneous hd-EEG-fMRI and outside-MR scanner hd-EEG available and were included in the present study (Table 1). Four of the 15 patients were diagnosed with juvenile absence epilepsy, three patients with childhood absence epilepsy, two patients with juvenile myoclonic epilepsy, three patients with isolated generalized tonic–clonic seizures, and three patients with GGE that could not be further classified. Except for one patient, all other patients were on antiseizure medication (ASM; mean number of drugs: 1.26, range: 0–2).

**Table 1:** Study population

	Patients with GGE	Healthy controls
Total (N)	15	16
Female n (%)	9 (60%)	7 (43.75%)
Age range	19–50	19–57
Mean (m)	32.5	31.77
Standard deviation (sd)	10.6	12.0

The measurements took place at the Universitätsklinikum in Tübingen. The local Ethics Committee of the Medical Faculty of the University of Tübingen gave approval for this study (ethical reference number: 646/2011BO1). The study was performed in concordance with the principles of the Declaration of Helsinki. All subjects gave informed consent before study participation.

### EEG and fMRI Data Acquisition

Hd-EEG acquisition was done using a 256-channel EEG system (Electrical Geodesics, Inc., Eugene, OR, USA) with a sampling rate of 1 kHz. The measurement was performed twice: first, outside the MR scanner room in supine position with eyes closed for 30 min (‘outside’) in a magnetically shielded room distant to the MR-scanner room. A second measurement was performed as a simultaneous hd-EEG-fMRI data acquisition within a 3 T scanner. Ten datasets were acquired using a Siemens MAGNETOM Trio, and 21 measurements were performed using a Siemens Prisma (Siemens AG, Erlangen, Germany). The measurement time varied across subjects; we acquired 10 min of data for five subjects, 15 min of data for 22 subjects, and four subjects were measured for 30 min. Also, a 3D T1-weighted 3D-MPRAGE sequence was acquired as high-resolution anatomical reference (TR: 2.3 s, TE: 3.03 ms, flip angle = 8°, voxel size: 1 × 1 × 1 mm). All functional MR data was acquired with a gradient-echo planar T2*-weighted sequence covering the entire brain (TR: 2 s, TE: 31 ms, voxel size: 3 × 3 × 4.2 mm, anterior–posterior (AP) phase encoding). To correct the functional MR images for distortions we also acquired 15 volumes with reversed phase-encoding (posterior–anterior) for each subject.

### Hd-EEG Processing

All preprocessing and further analysis steps on hd-EEG data were performed using Fieldtrip (https://www.fieldtriptoolbox.org/, version 20191127) running in Matlab (version 9.5.0.1298439 (R2018b) Update 7, Mathworks Inc.). The resting-state outside scanner hd-EEG data was filtered with a Butterworth band-pass filter with 1 Hz high pass and 70 Hz low pass. Also, a 50 Hz band stop filter was applied to account for line noise as well as its 100 Hz and 150 Hz harmonics. After that the data was downsampled to 250 Hz. We divided the continuous data in trials of 2-s lengths each. The data was visually inspected and noisy trials and/or trials artifacts contaminated by eye movements, blinks, cardiac, and muscle activity were rejected. Afterwards, we performed an independent component analysis to identify and remove components evincing electrocardiogram and blink/eye movement artifacts. In the patient group, any trial showing GSWD were manually marked by an experienced clinician and also removed together with the trials immediately before and after the GSWD trial.

The inside-MR scanner hd-EEG data was superimposed with the gradient artifact (GA) as well as the ballistocardiogram artifact. To remove those artifacts, we used algorithms implemented in the manufacturer’s software (Geodesic EGI tools, version: 5.4.2 (r29917)). In order to remove the GA, an average artifact subtraction method was applied. This method constructs an average GA template in each EEG channel and subtracts this template at the TR-trigger events from the raw EEG signal. The algorithm from Niazy et al. was used to detect and reject the BCG artifacts (Niazy et al. 2005). This algorithm applies a principal component analysis on the EEG sensor data time-locked to the detected cardiac events in the data. Principal components describing the BCG artifacts were chosen based on their amount of explained variance. This leads to the construction of an optimal basis set of the BCG artifact. Such OBS were used for adaptive artifact removal (Niazy et al. 2005). Further EEG data processing was performed in MATLAB identical to the outside-MRI EEG data, except for the separation of the continuous resting-state hd-EEG data in trials. Trials (2 s each) were synchronized by the MR-scanners TR trigger event, in order to achieve full correspondence between fMRI volumes and EEG trials.

### Forward Modelling and Source Analysis

For each subject, an individual cortical surface based on their anatomical MR image was reconstructed using FreeSurfer (https://surfer.nmr.mgh.harvard.edu, version 6.0.0) and subjected to SUMA (https://afni.nimh.nih.gov). The SUMA toolbox reduced the cortical surface of each subject to a fixed number of vertices (ld: 10, resulting in 2004 cortical vertices) based on the ‘fsaverage’ template (FreeSurfer). Furthermore, six subcortical nuclei (bilateral amygdala, hippocampus, thalamus, caudate, putamen and pallidum) were surface reconstructed using a total of 334 vertices from the fsaverage template. Those standardized subcortical vertices are transformed back to the individual anatomical space by using the inverted DARTEL transformation (DARTEL; SPM12; https://www.fil.ion.ucl.ac.uk/spm/software/spm12). This led to a total of 2338 vertices for each subject, with each vertex characterized to resemble a point-for-point anatomical correspondence for cortical and subcortical regions across all subjects. In the last step, each subject’s individual surface map was aligned to the CTF sensor space with the anatomical landmarks of fiducial positions (left/right preauricular point, nasion). For the subsequent source reconstruction, an individual boundary element model (BEM) with three layers of different conductivity (scalp: 0.33 *S*/*m*, skull: 0.004 *S*/*m*, brain: 0.33 *S*/*m*) was constructed using the ‘dipoli’ method implemented in Fieldtrip. The sensor time series were separated into common EEG frequency bands, namely delta 0–4 Hz (δ), theta 4–8 Hz (θ), alpha 8–12 Hz (α), beta1 12–20 Hz (β1), beta2 21–29 Hz (β2) and gamma 32–48 Hz (γ). Next, source projection was performed using dynamic imaging of coherent sources (DICS), a beamformer method, with $\lambda$ regularization of 5% for each frequency band separately (Gross et al. 2001). This approach was shown to be able to attenuate residual gradient and BCG artifacts that remained after averaged artifact subtraction methods (Brookes et al. 2008; Uji et al. 2021). The DICS method calculated a set of adaptive spatial filters, and the sensor level time courses were reconstructed in each vertex for each frequency band. Power estimation was calculated at each source vertex and for each frequency band. The source reconstruction was done separately for both conditions (inside and outside MR). For each subject, we randomly chose 200 wake-only trials, i.e. 400 s of data, to make all analyses comparable and to account for differences in the length of the measurement.

### EEG Functional Connectivity

For the electrophysiological data, FC analysis was performed by calculating the imaginary part of coherency (ImCoh) among all source-space time series and was calculated as noted below (Nolte et al. 2004):

1 $$Coherency_{xy} \left( f \right) = \frac{{S_{xy} \left( f \right)}}{{\sqrt {S_{xx} \left( f \right) \cdot S_{yy} \left( f \right)} }}$$

2 $$ImCoh_{xy} \;\left( f \right) = Imag\left\{ {Coherency_{xy} \;\left( f \right)} \right\}$$

where $S_{xy} \left( f \right)$ is the cross spectral density of the time series x and y at the frequency *f*. Further $S_{xx} \left( f \right)$ and $S_{yy} \left( f \right)$ are the power spectral densities of time series *x* and *y*. We took the absolute imaginary part of coherency $\left| {ImCoh_{xy} \left( f \right)} \right|$ as a measure for FC for each vertex. For each vertex in source space, we averaged all links (i.e. connectivity to all other vertices) as vertex-based connectivity (in the terminology of graph theory also called node strength). In addition, we calculated the global ImCoh as well as the global power values by averaging the vertex-based values for each subject in both inside-scanner as well as outside-scanner condition. We further calculated the relative difference $(\Delta \% )$ of global power/FC group between the inside- and outside-MR scanner condition’s group average in GGE and HC.

### fMRI Data Preprocessing

First, a correction of the data for slice-timing differences (SPM12, version 7487) was performed, followed by a head movement estimation using the Linear Image Registration Tool (FSL version 6.0.3, MCFLIRT) (Jenkinson 2002) as well as a distortion correction using the reversed phase-encoded scan (FSL, topup) (Andersson et al. 2003). The further processing steps were then performed using the CONN toolbox (http://www.nitrc.org/projects/conn, RRID:SCR_009550) (Whitfield-Gabrieli and Nieto-Castanon 2012). Outlier identification was done with the Artifact Detection Tools (ART) implemented in CONN and was set to ‘conservative’ settings. Thus, scans were marked as outliers if the observed global BOLD signal change was > 3 standard deviations and/or the amount of head movement was greater than 0.5 mm. In addition, in patients with GSWD, the corresponding fMRI volume and the following nine volumes (= 18 s) were marked as outliers in the fMRI data. A spatial co-registration of the averaged functional MR dataset to the anatomical T1 reference of each subject was performed. The anatomical T1 was segmented into tissue classes using the unified segmentation in SPM12 resulting in six tissue classes (grey-matter, white-matter, skull, scalp, cerebrospinal fluid and others) and a non-linear transformation of each individual T1 scan to the MNI152 T1 reference. This transformation matrix was used to spatially normalize all fMRI volumes and the segmentation masks to the MNI152 space. The grey matter class was further parcellated into different atlas regions according to the Harvard–Oxford Cortical Atlas, and network regions were created by grouping atlas nodes. The fMRI data was further smoothed using a Gaussian kernel of 8 mm full-width at half maximum. To further control for spurious effects of head motion, and cardiac and respiratory rhythms, each subject’s data was corrected by partial regression in CONN removing the following confounds: six motion parameters (three rotation and three translation parameters, based on the MCFLIRT rigid body transformation), subject-specific confounding factors that modeled nuisance signals within the white matter and CSF segmentation masks, each with three PCA parameters that were used as temporal covariates. No global signal regression was applied, to avoid the introduction of spurious connectivity (Murphy et al. 2009; Weissenbacher et al. 2009). Finally, the BOLD time series was band-pass filtered with 0.008–0.09 Hz.

### Seed-Based Functional Connectivity Analysis of EEG and fMRI

A seed-based functional connectivity (sbFC) analysis was performed in both modalities, choosing seed-regions from the grey matter parcelling based on the atlases included in the CONN-toolbox. Seed regions were chosen based on a priori information from previous studies that reported differences in GGE versus HC comparisons. Namely, one network seed at the medial prefrontal cortex, the left and right lateral–parietal default mode network (DMN-LP L/R), and an atlas seed at the precuneus cortex (PCC) (Luo et al. 2011; McGill et al. 2012). Also the intracalcarine cortex (left and right) was used as an atlas seed in both hemispheres, since they are located close to the PCC region and are also associated with the default mode network. Lastly, the left and right thalamus atlas regions were added to the list of seeds of interest, given their known involvement in GSWD-associated as well as resting-state network changes in GGE (Gotman et al. 2005; Hamandi et al. 2006; Ji et al. 2015; McGill et al. 2014; Wang et al. 2011). Seed-based FC (Pearson correlation) in fMRI was performed in MNI voxel space between each seed region and every other voxel in the brain, defined using the default standard ‘brainmask’ in CONN, which includes both cortical and subcortical voxels. Additionally, the subject-specific seed-based functional connectivity maps were transformed and resampled from 3D MNI space to the surface vertices (SUMA and subcortical surface) to allow direct spatial comparison to the hd-EEG sbFC maps.

Similar to fMRI, a seed-based FC in hd-EEG was calculated using the imaginary part of coherency (Nolte et al. 2004). To this end, all surface vertices were labeled according to the CONN atlas via the individual spatial transformation matrix generated by SPM. We grouped vertices with identical region labels and chose the same regions as seeds as we did for the fMRI seed-based analysis to calculate sbFC on EEG data for each subject.

### Permutation Analysis of Linear Models

The non-parametric statistics toolbox ‘Permutation Analysis of Linear Models’ (PALM) was used to calculate group differences between patients with GGE and healthy controls for power and FC in the EEG as well as seed-based FC in both modalities (https://fsl.fmrib.ox.ac.uk/fsl/fslwiki/PALM, version: alpha103) (Winkler et al. 2014).

Briefly, using PALM, general linear models were run for each vertex and one at the global level, with additional covariates of subject age and gender. A comparison was made using a one-tailed t-contrast for the groups on a vertex-, and voxel-level, as well as at the global level, separately for each frequency band. This was determined based on the results of our previous studies (Stier et al. 2022). The resulting p-values were generated based on an estimate of the empirical distribution of t-statistics under the null hypothesis by 20,000 random permutations of the subjects. In the vertex-, and voxel-based analysis, we corrected for multiple comparisons at the cluster level using a threshold-free cluster enhancement with default parameters set by PALM (height = 2, and extend = 1) (Smith and Nichols 2009). In order to evaluate the hypothesis that the same pattern can be found for inside-MR scanner EEG data, we performed the same comparison (GGE versus healthy controls) for both conditions (outside MR, inside MR). Furthermore, we tested the differences in global power and FC depending on the measurement condition, and an interaction with the group assignment (patients with GGE and controls) using a 2-way ANOVA (2-groups, and 2-levels per subject) in PALM and applied false discovery rate (FDR) (Benjamini and Hochberg 1995) correction across frequency bands. We also applied FDR correction for the ${-}log_{10} \;p$-values from the group differences in global power and functional connectivity across frequency bands. We also reported both uncorrected values and ${-}log_{10} \;p$-values that survived FDR correction. Statistically significant group differences on fMRI seed-based FC were derived from the sbFC maps in 3D MNI space, as well as from the sbFC resampled on the surface vertices of each subject. Statistical comparisons were carried out with 20,000 random permutations. For each permutation, a general linear model was estimated including sex and age (and for fMRI data, the number of scan volumes) as covariates of no interest. To correct for multiple comparisons across space, familywise error correction (FWE) was applied to p values of all vertex- and voxel-based tests, represented as ${-}log_{10} \;p$. Statistical significance level was defined for values > 1.3 (corresponding to *p* < 0.05 [corrected]). Additionally, effect sizes (Cohen’s *d*) was calculated for each statistical test, defining effect sizes of *d* = 0.2 as small, *d* = 0.5 medium and *d* = 0.8 as a large effect size (Cohen 1992). In order to quantify the spatial similarity between EEG group difference maps (${-}log_{10} \;p$-value maps without a threshold) of inside and outside MR-scanner for vertex-based power and functional connectivity we calculated Pearson correlation with corresponding p-values, which were FDR corrected across frequency bands.

### Cross-Modal Spatial Relation

We compared the group differences (GGE > HC) for each seed-based functional connectivity map from fMRI data. Only seeds that showed a suprathreshold group difference (p_FWE_ < 0.05) were considered further for cross-modal spatial correlation analysis. In addition, we only considered EEG frequency bands that showed a statistical significant group difference (p_FWE_ < 0.05) in vertex-based functional connectivity for further analysis.

Pearson correlation coefficients were used to estimate the spatial concordance between EEG and fMRI group difference maps. To control for chance findings, we generated surrogate datasets by shuffling the EEG sensors of inside-MR hd-EEG data of each subject. This was done 50 times yielding 50 different surrogate datasets for each subject. Identical to the analysis of the real data, the surrogate datasets underwent source analysis and the results were used to calculate a seed-based FC map for each statistically significant fMRI seed-based FC analyses in each in frequency band that showed statistical significance in the group comparisons of vertex-based FC. After that, a group comparison was calculated via PALM. This was done for 500 surrogate permutations; in each permutation, surrogate data was chosen randomly for every subject from the 50 available surrogate datasets. This resulted in 500 FWE-corrected ${-}log_{10} \;p$-value maps for each frequency band that showed statistical significance in the group comparisons of vertex-based FC and each statistically significant fMRI seed-based FC analyses. The cross-modal correlation to the fMRI ${-}log_{10} \;p$-value map was then also performed for all ${-}log_{10} \;p$-value maps based on surrogate datasets. Only cross-modal correlation values of the real data that surpassed the 95th percentile of the surrogate data were considered significant. The resulting p-values were multiple comparison corrected using false discovery rate (Benjamini and Hochberg 1995).

## Results

A statistical group comparison of HC and patients with GGE showed no significant differences for age or sex between the groups (age: *p* = 0.456 [t-test]; sex: *p* = 0.37 [$\chi^{2}$ test]). On average 31% of the trials (133 ± 48) for the inside MR-scanner condition and 29% (283 ± 138) for the outside MR-scanner condition we rejected due to artifacts in the data. In total five trials with GSWD were identified and rejected in four different patients in the inside MR-scanner condition. For the outside MR-scanner condition five trials with GSWDs in five different patient) were marked and also rejected.

### Global Power

**Fig. 1:**
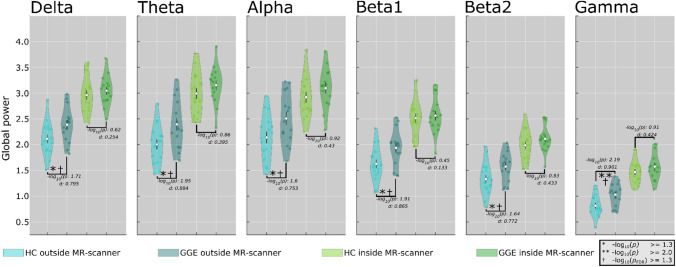
Global power depicts in violin plots. Distribution of global power for patients with GGE and HC in the inside- (green colors) and outside-MR scanner condition (blue colors) for each frequency band. Violin plots show subject-specific data points, the density of the group data, group means, and standard error of the means for global power in each frequency band. Asterisks mark statistically significant differences between groups (GGE > HC) (*${-}log_{10} \;p$ > = 1.3), and † mark statistically significant differences that survived FDR correction (*${-}log_{10} \; p$_FDR_ > = 1.3). Note that significant group differences and large effect sizes were found for all frequency bands for the outside condition only (GGE > HC), and all survived multiple comparison correction via FDR. The significant group differences and effect sizes for the inside condition are smaller compared with the outside condition in all frequency bands

For global power, significant group differences (GGE > HC) were found in each frequency band for the outside-MR scanner condition (${-}log_{10} \; p$ > 1.3), as expected from previous work. For the inside-MR condition, an overall increase of global power was observed for both groups (HC and GGE) in all frequency bands. The relative differences $(\Delta \% )$ between the group means from outside to inside condition were increased in both groups and all frequency bands (Δ%(delta) = HC: 40%, GGE: 28%; Δ%(theta) = HC: 49%, GGE: 32%; Δ%(alpha) = HC: 37%, GGE: 24%; Δ%(beta1) = HC: 55%, GGE: 32%, Δ%(beta2) = HC: 49%, GGE: 34%; and Δ%(gamma) = HC: 80%, GGE: 53%) (Appendix: Table 3). The difference of global power between conditions (inside > outside) was highly significant (*p*_FDR_ ≤ 0.001, *d* > 1.2) in all frequency bands. However, the interaction of inside–outside condition and group difference was not significant after error correction (*p*_FDR_ > 0.05, *d* < 0.25) (Appendix: Table 2). The group differences between HC and GGE were not statistically significant in any frequency band for the inside-MR condition (Fig. 1). Equally, the effect sizes were smaller in the inside- (*d*: 0.13–0.43) compared with the outside- (*d*: 0.75–0.96) MR condition.

### Global Functional Connectivity

**Fig. 2:**
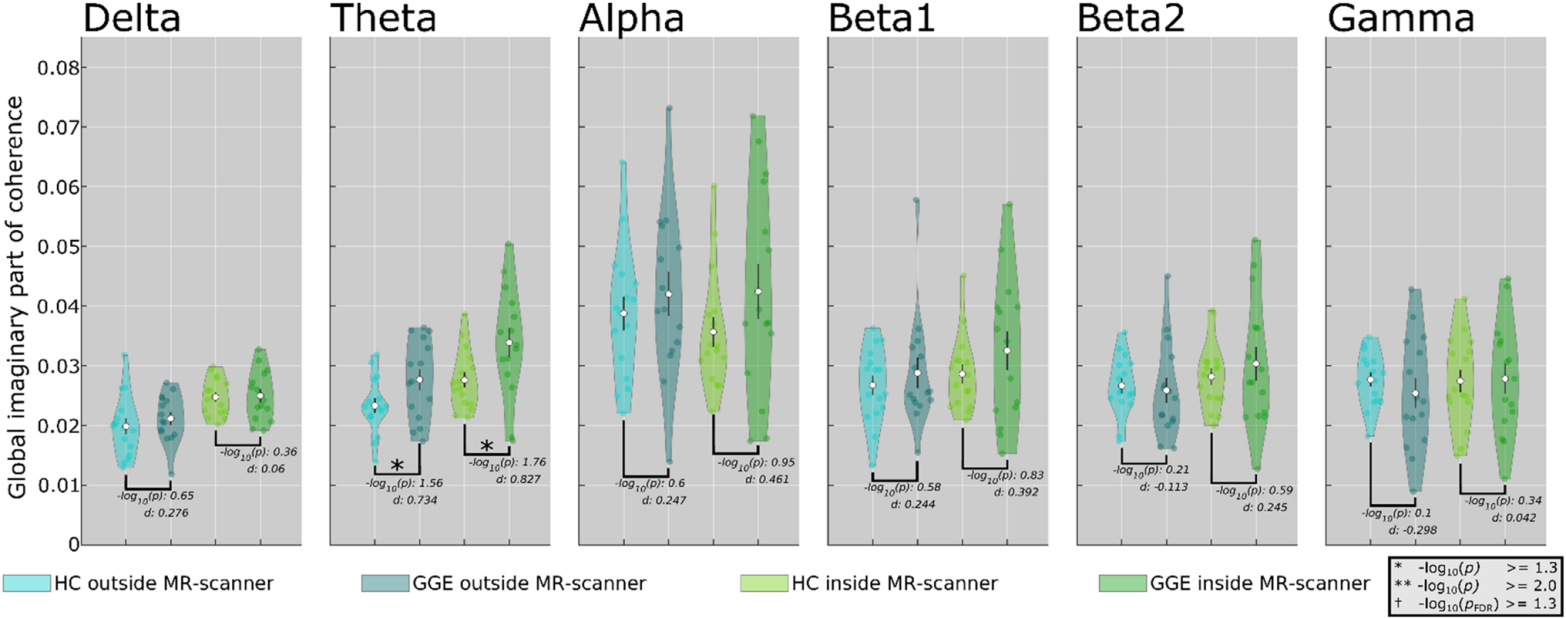
Global imaginary part of coherency depicted in violin plots. Distribution of global functional connectivity (ImCoh) for patients with GGE and HC in the inside- (green colors) and outside-MR scanner condition (blue colors) for each frequency band. Violin plots show subject-specific data points, the density of the group data, group means, and standard error of mean for global connectivity in each frequency band. Asterisks mark statistically significant differences between groups (GGE > HC) (*${-}log_{10} \;p$ > = 1.3). Note that significant group differences (${-}log_{10} \;p$) were found for the theta (*${-}log_{10} \;p$ > 1.3) band only (GGE > HC) for both conditions. However, this difference would not surpass FDR correction for multiple comparisons. The effect size values for the inside condition were higher compared with the outside condition in all frequency bands, but delta

The change in the group means for FC varied across frequencies, and no common pattern was evident. The relative difference (Δ%) between the GGE and HC group means of global FC was different in all frequency bands from outside to inside condition (Δ%(delta) = HC: 25%, GGE: 18%; Δ%(theta) = HC: 18%, GGE: 22%; Δ%(alpha) = HC: − 8%, GGE: 1%; Δ%(beta1) = HC: 7%, GGE: 13%, Δ%(beta2) = HC: 6%, GGE: 17%; and Δ%(gamma) = HC: − 1%, GGE: 10%) (Appendix: Table 4). The difference of global FC between conditions (inside > outside) was highly significant for two frequency bands (delta: *p*_FDR_ < 0.001, *d* ≈ 1.0; theta: *p*_FDR_ < 0.01, *d* ≈ 0.73). No significant interaction effect was observed (*p*_FDR_ > 0.05, *d* < 0.05) (Appendix: Table 2). Statistically significant group differences (GGE > HC) were only found in theta for both conditions (outside-MR condition ${-}log_{10} { }\;p$ = 1.56; inside-MR scanner condition ${-}log_{10} { }\;p$ = 1.56). For both conditions, a medium to large effect size (outside: *d* = 0.734; inside *d* = 0.827) was found in theta. As opposed to power, we even found an increase in ${-}log_{10} { }\;p$-values and statistical effect sizes (*d*) from outside- to inside-MR scanner group differences in all frequency bands, except for the delta band (Fig. 2).

### Vertex-Based Analysis of Power

**Fig. 3:**
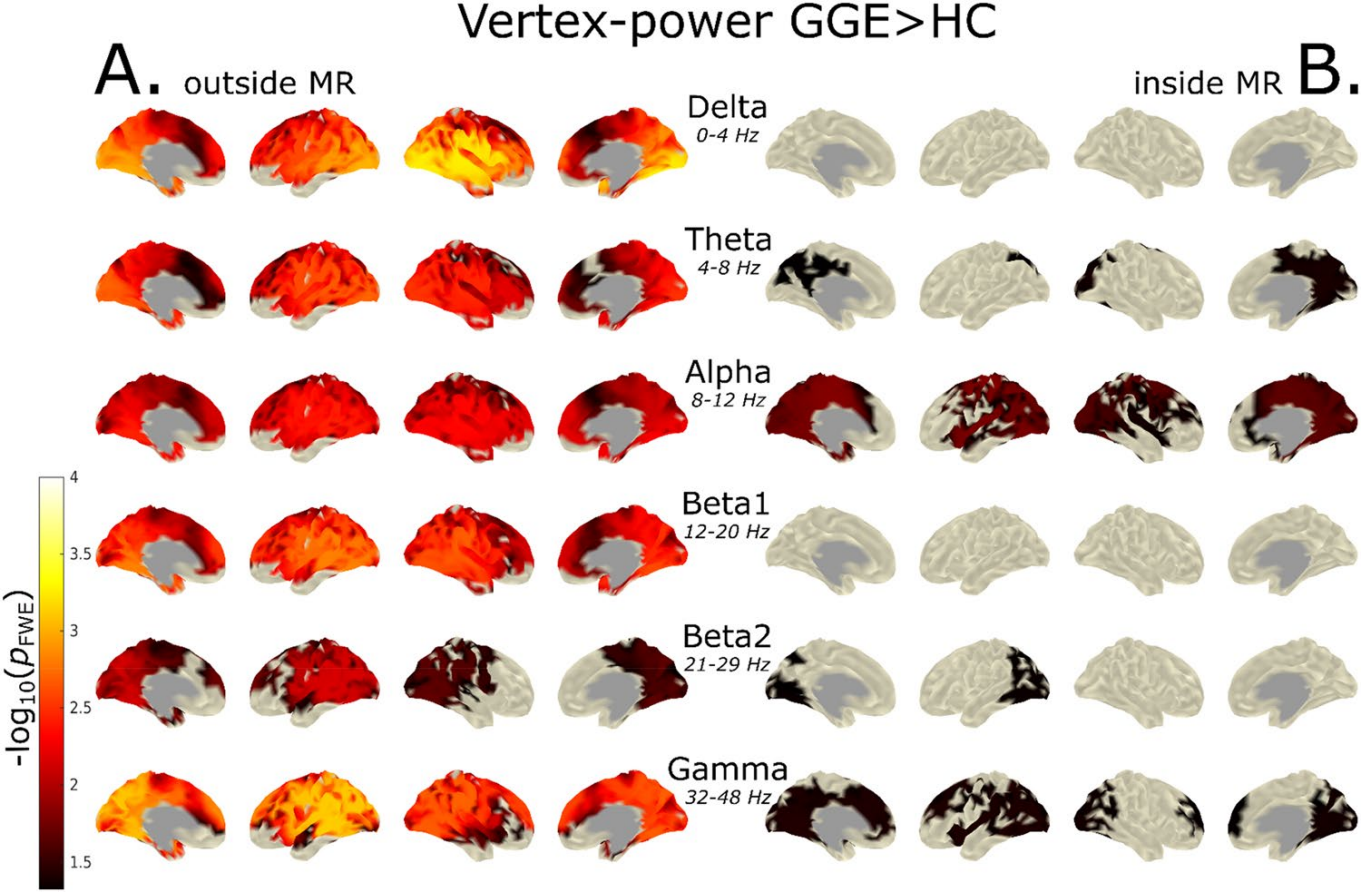
Vertex-power group analysis (GGE > HC) of inside- and outside-MR scanner conditions. Group contrast between patients with GGE and heathy controls (GGE > HC) of vertex-based power in each frequency band calculated using PALM. In row **A** the statistical contrast of the outside-MR data based on vertex power is shown and row **B** illustrates the inside-MR scanner condition. Note that the ${-}log_{10} \;p$-values in the inside-scanner condition were smaller than in the outside condition but they still surpassed the statistical significance threshold of ${-}log_{10} \;p$ > 1.3 and attained significance in fewer but identical regions. Note that the ${-}log_{10} \;p$-values in the inside scanner condition are smaller in comparison to the outside condition, but show high spatial similarity (*r* > 0.85, *p*_FDR_ < 0.001) between measurement conditions

The statistical group analysis of vertex-based power in the outside-MR scanner condition showed a widespread significant increase for patients with GGE in all frequency bands (${-}log_{10} { }\;p$ > 2) (Fig. 3A). An occipital/precuneus and lateral–parietal focus of power increase in patients with GGE was found in all frequency bands with the highest significance in the delta and gamma bands (${-}log_{10} { }\;p$ > 3). In addition, the medial frontal regions showed greater power in patients with GGE in all frequency bands, except beta2.

The topographies of the significant increase of power in patients with GGE in each frequency band are similar to the results of our previous work. A decrease in ${-}log_{10} \;p$ values was evident in all frequency bands.

Contrary to this, we found a strong reduction in the group differences (GGE > HC) for the inside-scanner condition (${-}log_{10} { }\;p$ ≈ 1.3–2.0) in all frequency bands (Fig. 3B). After correction for multiple comparisons the group differences in the delta and beta1 frequency bands did not surpass the significance threshold. The greatest group difference was found in the alpha band (${-}log_{10} { }\;p$ ≈ 2), while the other frequency bands showed smaller, but statistically significant group differences (${-}log_{10} { }\;p$ ≈ 1.3–1.5). An occipital/precuneus and lateral–parietal focus of power increase in patients with GGE was found in most frequency bands.

Although both significance, as well as effect size decreased for the inside condition and reached significance in fewer but identical regions, the increased power in patients with GGE in the posterior regions of the brain was consistent. Further, the group difference maps of inside and outside MR-scanner for vertex-based power showed high spatial similarity in all frequency bands (*r* > 0.85, *p*_FDR_ < 0.001).

### Vertex-Based Functional Connectivity Analysis

**Fig. 4:**
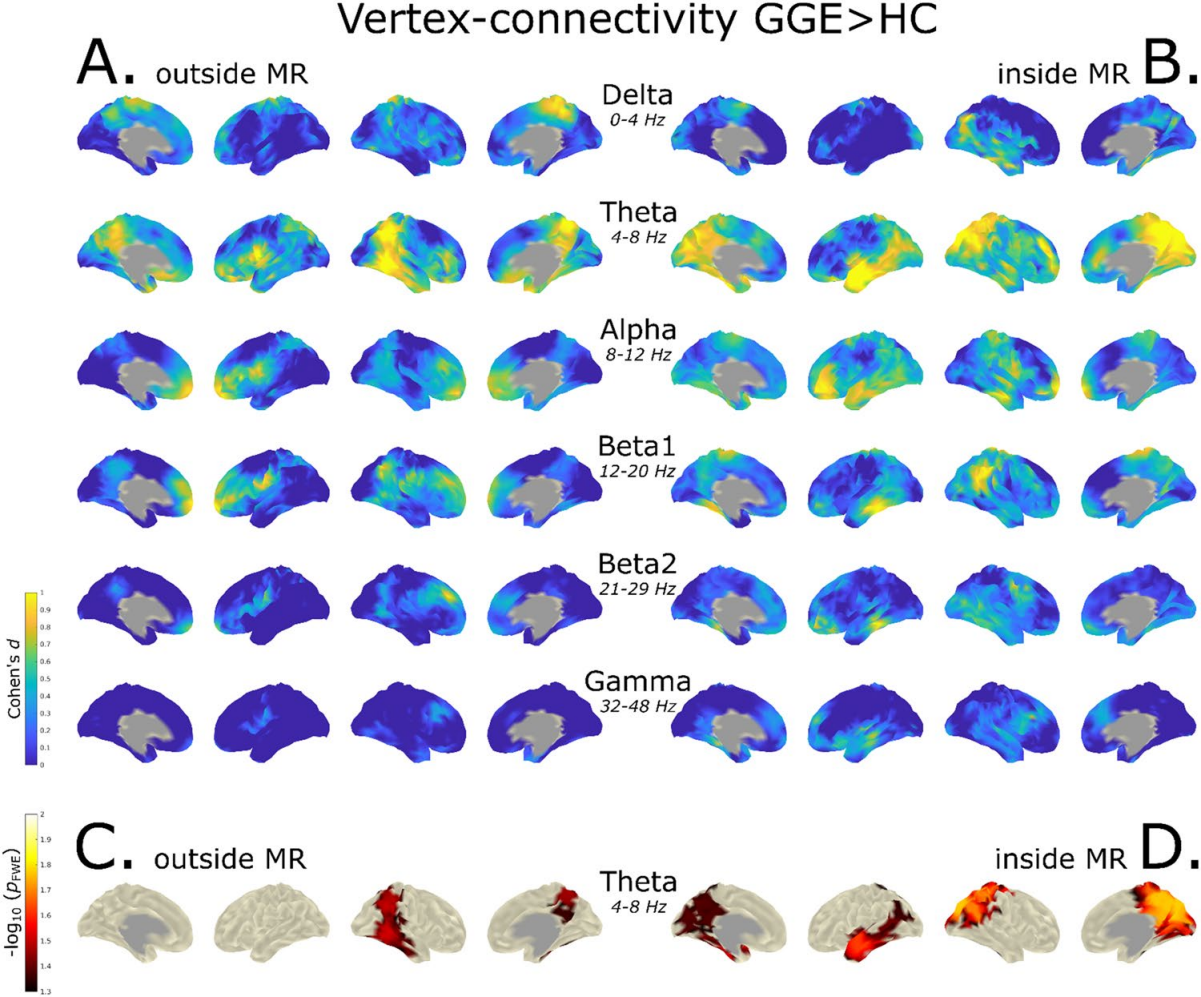
Vertex-connectivity group analysis (GGE > HC) of inside- and outside-MR scanner conditions. Standardized effect sizes (Cohen’s *d*) for group differences (GGE > HC) for the outside- (**A**) and inside-MR condition (**B**). In addition, the significantly different vertices (p < 0.05, FWE corrected) are shown for the outside- (**C**) and inside-MR condition (**D**). Note that the strongest effects were found in the theta band, and the group differences were only significant in this frequency band after FWE-correction. Interestingly the significance was higher (${-}log_{10} \; p$ ≈ 1.8) and the effect size stronger (*d* > 1) for the inside-MR scanner condition. High spatial similarity was found for the statistical group difference ${-}log_{10} \; p$-value maps for theta (*r* = 0.55, *p*_FDR_ < 0.001)

In the outside- as well as inside-MR scanner condition, the group comparison (GGE > HC) surpass the cutoff of ${-}log_{10} \;p$ > 1.3 after statistical correction only in the theta frequency band. Hence, to better visualize the effect of the inside-MR scanner measurement condition in all frequency bands, we compared the group differences between the conditions on the basis of effect sizes (Cohen’s *d*) (Fig. 4A, B).

The group difference effect sizes (*d*) of vertex-based functional connectivity for GGE > HC in the outside condition were largest in the theta band (*d* > 1), located in the right lateral parietal cortex (DMN lateral–parietal right) region and in the PCC in both hemispheres (Fig. 4). We also found large effect sizes (*d* > 1) in the left superior temporal region. In the delta frequency band, large effect sizes (*d* > 1) were found in the fronto-parietal region, with stronger effects in the right hemisphere. In alpha and beta1 medium to large effect sizes (*d*: 0.75 to > 1) were found in the medial prefrontal cortex region. A similar distribution was detected in beta2, but with medium effect sizes (*d* *≈ *0.5), which were smaller for gamma (*d* *≈ *0.35). The topography of the effect size is similar to the results of our previous work of the same cohort with corresponding group differences in delta, theta and alpha.

For the inside-MR condition, we found the largest effect sizes in the theta band (*d* > 1) with a focus on the PCC region, and the same effect size pattern (*d* *≈ *0.8–1.0) in the left region of the hemisphere. In beta1 medium effect sizes (*d* *≈ *0.3–0.4) with an effect size cluster at the right lateral–parietal region with large effect sizes (*d* *≈ *0.85–1.0) were found. In beta2 a similar distribution was found, but with considerably smaller effect sizes than in beta1 (*d* *≈ *0.4–0.55), and even smaller in gamma (*d* *≈ *0.3–0.4).

Across conditions, we found the largest effect sizes (*d* > 0.95) and coherent distributions in the PCC region in the theta band. Effect sizes showed an increase and a wider spatial extension compared with the outside-scanner results. In addition, the right lateral parietal region had large effect sizes in both conditions. However, in the inside-MR scanner condition the focus was found to be more posterior and superior. The large fronto-temporal cluster seen on the effect size maps of the left hemisphere for the outside-MR scanner condition was only located in the left temporal lobe in the inside-MR scanner results. Between conditions a minor increase in effect sizes was visible in all frequency bands, except for delta.

Statistical significance was only found in the theta band after FWE-correction in both conditions with greater group differences for GGE > HC (Fig. 4C, D). Inside and outside MR ${-}log_{{10{ }}} \;p$ maps showed relevant spatial similarity (*r* = 0.55, *p* < 0.001) between both conditions in the theta band, but no relevant spatial similarity was observed in the other frequency bands.

For the outside-MR scanner condition statistically significant FC increases were found in patients with GGE in the right lateral–parietal to temporal cortex (${-}log_{{10{ }}} \;p$ ≈ 1.5–1.6) and the right PCC region (${-}log_{{10{ }}} \;p$ ≈ 1.55) (Fig. 4C). The inside-MR scanner condition showed FC increases in patients with GGE in the left temporal lobe (${-}log_{10} \;p$ ≈ 1.6) and PCC region in both hemispheres (left: ${-}log_{10} \;p$ > 1.4; right: ${-}log_{10} \;p$ > 1.75) and (Fig. 4D).

### fMRI Seed-Based Functional Connectivity

**Fig. 5:**
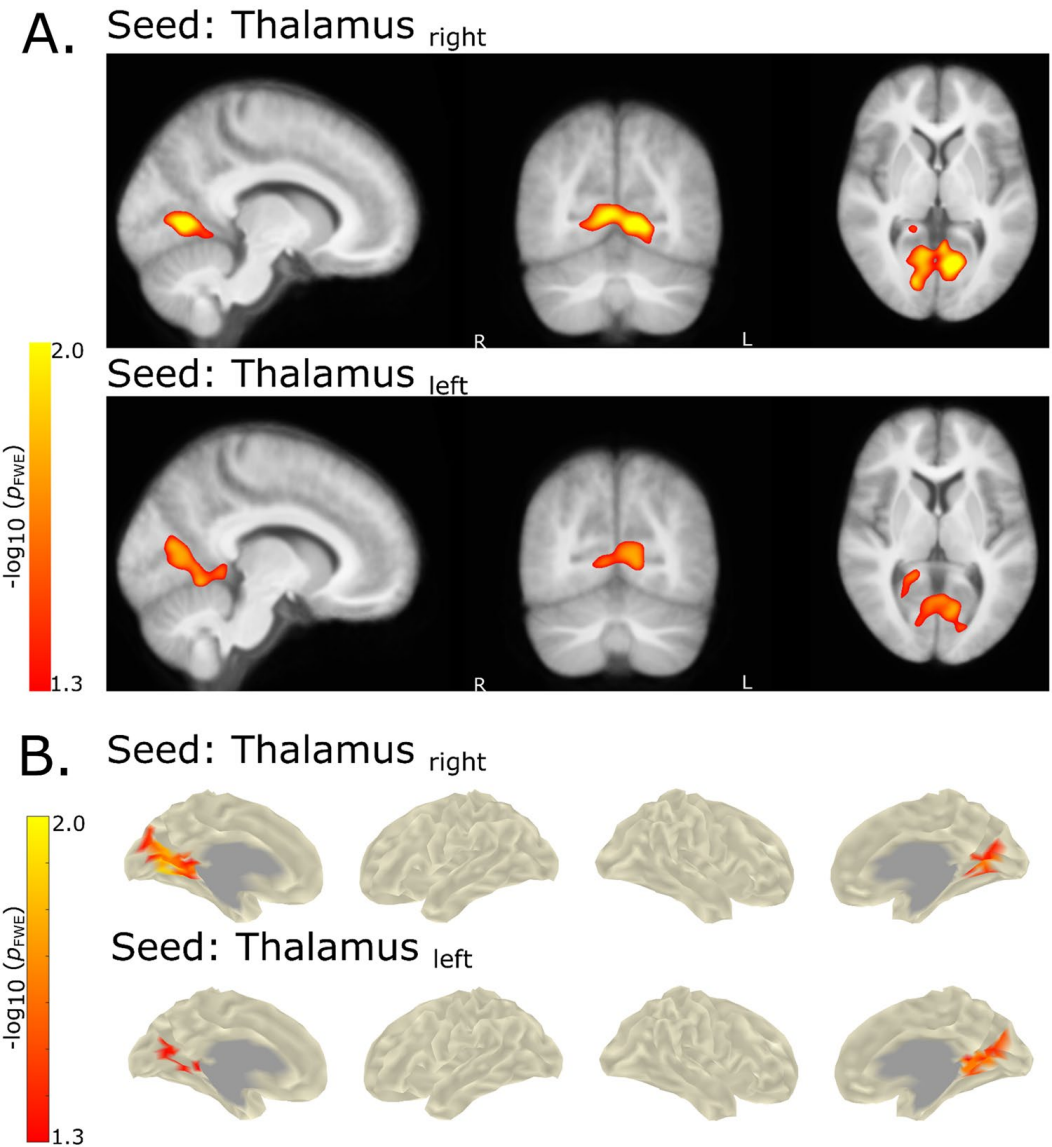
Group differences (GGE > HC) of seed-based FC derived from fMRI data. Statistically significant increases of seed-based FC for GGE > HC for thalamus seed regions. **A** Based on voxel correlation maps derived from CONN and **B** derived from transformed and resampled correlation maps on surface vertices. Note that both approaches show nearly identical increases in sbFC for GGE > HC from thalamus seed regions to regions of the PCC. Moreover, no decreases, but only increases in sbFC were found in patients with GGE

We found a statistically significant increased sbFC in patients with GGE for the thalamus seed regions and posterior cingulate/precuneus areas, both for a voxel as well as surface-based approach (Fig. 5). This group difference was slightly stronger for the left thalamus seed region but both seeds surpassed the FWE-corrected cutoff of ${-}log_{10} \; p$ > 1.3. No other seed regions showed a statistically significant increase or decrease in patients with GGE versus controls.

### Spatial Relation of fMRI and EEG Seed-Based Functional Connectivity

**Fig. 6:**
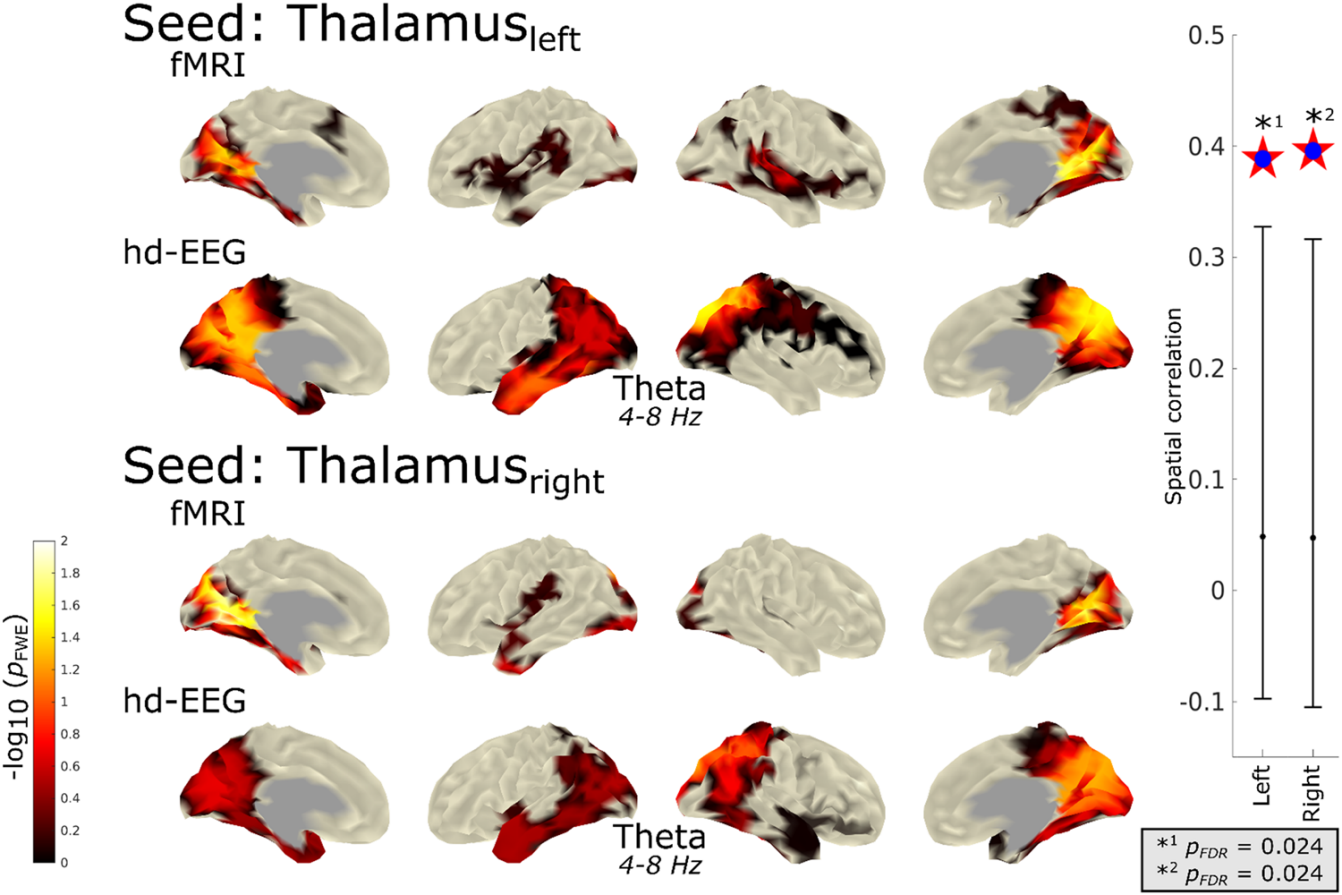
Cross-modal spatial correlation of sbFC group contrasts results of GGE > HC in fMRI and EEG. This figure shows the spatial topography of seed-based functional connectivity for left and right thalamus seed regions for fMRI and EEG. On the right, the distribution of the observed spatial correlation versus the surrogate data is shown including error bars representing the 95th percentiles. It is evident that the overserved correlation between EEG and fMRI was significantly higher than surrogate data (left: *p*_*FDR*_ = 0.024; right: *p*_*FDR*_ = 0.024). Note: for better visualization of the full topography of the maps, no cutoff was used. Values below 0.05 are shown as transparent. Statistical significance of spatial correlation was assessed via the comparison to surrogate data

Further, we aimed to link the fMRI and EEG seed-based FC group differences. Figure 6 shows the side-by-side surface plot of fMRI (based on correlation) as well as EEG seed-based FC (based on ImCoh) group differences. The topography was similar, however, the group differences were more extended for EEG. A spatial correlation was calculated between EEG and fMRI (left ≈ 0.39; right ≈ 0.4). These correlation values were significantly higher (left: *p*_FDR_ = 0.024; right: *p*_FDR_ = 0.024) than the correlation values derived from the surrogate EEG data, indicating that these correlations are stronger than what could be expected by chance.

Group differences of sbFC from the right thalamus seed region did not surpass the statistical threshold of ${-}log_{10} \;p$ > 1.3 but showed a stronger spatial correlation across modalities than the left thalamus seed region. The topographies of increased sbFC in patients with GGE in the occipital, lateral–parietal and temporal regions resemble the increase in vertex-based FC in the theta band.

## Discussion

We analyzed resting-state EEG data of patients with GGE and healthy controls under two measurement conditions: inside (with simultaneous fMRI) and outside the MR scanner. We showed that the analysis of power is strongly affected by the within-MR conditions, whereas imaginary part of coherency was less influenced. Moreover, seed-based FC analysis was suitable for simultaneous EEG-fMRI revealing a consistent multi-modal functional connectivity increase for GGE between thalamus and posterior cingulate cortex, indicating that the thalamo-cortical loops may play a role in the increased resting-state connectivity observed in GGE.

Compared to our previous work, we observed a similarly significant power increase in patients with GGE in all frequency bands in the outside-MR scanner condition. Further, we were able to reproduce the statistically significant group difference for outside-MR scanner global FC in the theta band but with slightly lower effect size and statistical significance. In addition, we found lower statistical significance at vertex-level in frequency bands delta and alpha compared with previous results. The observed lower statistical significance is likely due to a smaller number of subjects available in the present study than in the previous analysis (GGE: + 8 subjects, HC: + 16 subjects) (Stier et al. 2022). It is also likely that the altered topological representation of group differences in patients with GGE in this study is related to the cohort differences of the two analyses.

Furthermore, to allow for synchrony with the fMRI TR we used 2-s trials for both conditions. It has been shown that the length of EEG data trials is affecting the estimation of some FC metrics (Brookes et al. 2011), which may have an impact on the group-level results in this study. Yet, previous work has shown that 5–6 min of data is sufficient for a reliable analysis of FC at rest (Marquetand et al. 2019; Rolle et al. 2022). In this study, we used more than 6.5 min to compensate for the shortening of the trial duration. Besides technical reasons, it is also plausible that the different measurement environment influenced the subject’s physiological state. The confined surroundings in an MR scanner with its loud noise may lead to a different mental state in the subjects.

A highly significant increased global power was observed in all groups and frequency bands for the inside-MR condition, accompanied by a reduced GGE-control group differences. However, the interaction of these two factors was not significant after error correction.

Although common GA and BCG artifact removal methods were applied, residual noise could alter the power spectral density of the sensor timeseries (Allen et al. 1998). Since the signal-interaction of different artifact sources (including subject motion) is complex, a complete artifact removal is hardly possible (Jansen et al. 2012). Therefore, it is most likely that noise residuals remain embedded in the data. The increase in signal amplitude (Allen et al. 1998) and spectral power in the artifact-corrected parallel EEG-fMRI data was observed at a similar extent in previous work (Abreu et al. 2016; Debener et al. 2007; LeVan et al. 2013; Masterton et al. 2007; McIntosh et al. 2021; van der Meer et al. 2016). Consequently, global and vertex-based results derived from EEG power for inside-MR scanner must be interpreted with caution, despite the topographic similarity of group differences (GGE > HC) at vertex-level.

Higher signal power is associated with increased phase-based FC metrics (Daffertshofer and van Wijk 2011; Moon et al. 2015). Therefore, the power increase seen at global- as well as at vertex-level could have influenced the FC calculation and have resulted in higher FC values. Even though the imaginary part of coherence is based solely on phase-shifted connectivity, it is still amplitude biased, e.g. via leakage of applied inverse methods (Sekihara et al. 2011). This fact, in combination with noisier data, might also lead to a poorer estimation of phases, and therefore lead to deviations in the topological representation of FC values in the inside-MR scanner condition. However, temporally synchronized artifacts in the data should have less effect on this metric, since the imaginary part of coherence is focused on phase-shifted connectivity (Nolte et al. 2004), which could explain why we found largely similar results for the statistically significant difference between GGE and HC in theta for the inside- and outside-MR conditions. This is supported by the finding of existing peaks in the Fourier spectrogram in the theta band for inside and outside MR-scanner data sets, as well as MR-artifact uncorrected data sets. Despite the theta frequency band spatial correlation between inside and outside MR-scanner condition was found to be rather small (see Supplementary Fig. 8). From a previous study, it is known that compared to power, ImCoh is a relatively unstable metric in repeated measurements. This means that the results for ImCoh of a measurement cannot be an exact representation of the results of a previous measurement, even for the same cohort (Marquetand et al. 2019).

Our findings for the seed-based fMRI connectivity are in line with an earlier study, that found increased FC from the thalamus to posterior cingulate cortex regions in resting-state fMRI data (Ji et al. 2015). In contrast to other studies, we did not observe decreased seed-based FC in other DMN brain regions in patients with GGE (Luo et al. 2011; McGill et al. 2012). Various factors can be responsible for the differing results, e.g. the composition of our cohort with mixed GGE syndromes, and also technical features. Other studies used different spherical seed radiuses from 6 to 10 mm (Luo et al. 2011; McGill et al. 2012) and/or 26-neighborhood voxels around the seed position (Luo et al. 2011), while we used an atlas-based definition for seed regions. Further, McGill et al. (2012) applied global signal regression on their resting-state fMRI data before doing FC analysis, as the global signal in functional MRI time courses was often considered a nuisance effect. In our analysis the global signal regression was omitted, because this step might introduce spurious connectivity into the data and there is an ongoing debate in the field if this should be included or not (Murphy et al. 2009, 2013; Weissenbacher et al. 2009; Saad et al. 2012; Murphy and Fox 2017; Liu et al. 2017; Aquino et al. 2020).

Concerning the EEG seed-based connectivity, we provided evidence of an increased connectivity between thalamus and posterior cingulate cortex region in GGE patient’s theta band derived from EEG with a parallel fMRI measurement. Multiple previous studies described FC increases (review: Faiman et al. 2021), in particular in the theta band in patients with GGE using outside-MR scanner EEG/MEG data (Clemens et al. 2011; Douw et al. 2010; Elshahabi et al. 2015; Li Hegner et al. 2018; Routley et al. 2020; Stier et al. 2021, 2022). In this work, we observed a strong similarity between vertex-based sbFC and FC results in the theta band, indicating that thalamo-cortical loops may play a role in the generation of this increased FC at rest. However, for our analysis we cannot identify the underlying causes of this network alteration in GGE patients’ theta band. Nevertheless, in addition to the already known involvement of the thalamus in the development and propagation of GSWD as reported in MEG studies (Westmijse et al. 2009; Miao et al. 2014), our results indicate a stronger continuous involvement of the thalamus in resting-state/default mode brain networks in patients with GGE in the theta band. Future studies are required to investigate the thalamo-cortical connectivity in patients with GGE in more detail.

Previously, the FC increase in GGE between thalamus and posterior cingulate cortex was only shown in an fMRI study (Ji et al. 2015). Our work demonstrates that this network alteration in GGE can be extracted from both modalities. Further, the spatial cross-modal coherence of thalamo-cortical network alterations in GGE at rest indicates that these changes are expressed at both levels: neuronal electrical activity and vascular changes.

## Limitations

Due to the small sample size and a mixed cohort of patients with GGE, we were unable to identify GGE sub-syndrome specific effects or detect their influence on the analysis. Further, although we utilized well-established techniques to remove the GA and BCG artifacts in the EEG data (Niazy et al. 2005) more elaborated methods may lead to different results. For example, artifact correction using carbon-wire loops during EEG-fMRI acquisition might have improved the artifact rejection and thus led to less noisy EEG data (van der Meer et al. 2016). In addition, other approaches of removing MR artefacts may give different results, e.g. using the online method proposed by Masterton et al. (2007). In addition, we cannot evaluate the impact of our equipment on our measurements because no alternative compatible equipment was available for direct comparison. Finally, we used measurement time for parallel EEG-fMRI of 10–30 min. Longer measurement times may lead to different and/or more stable results. However, similar fMRI studies used 6–8 min of data for their analysis (Ji et al. 2015; Luo et al. 2011; McGill et al. 2012; Wang et al. 2011), which is similar to the data amount used in this works analysis.

## Conclusion

Overall, we showed that EEG resting-state network analysis is suitable for inside-MR data. After conventional EEG artifact cleaning, the power estimates inside the MR scanner were significantly increased. Nevertheless, similar spatial topography and general direction of group differences remained. Phase-lagged coherence (ImCoh) was less affected by the inside-MR condition and yielded group difference significances comparable to those of the outside-MR scenario. Moreover, with seed-based FC we found increased connectivity from the thalamus to the precuneus cortex for GGE in fMRI and EEG theta band. This indicates that both modalities probe directly related processes and networks.

## Supplementary Information

Below is the link to the electronic supplementary material.

Supplementary file1 (DOCX 7609 KB)

## Data Availability

The data that support the findings of this study are available from the corresponding author upon reasonable request.

## Abbreviations

ASM: Antiseizure medication
BCG: Ballistocardiogram
BEM: Boundary element model
DMN: Default mode network
ECG: Electrocardiogram
FC: Functional connectivity
fMRI: Functional magnetic resonance imaging
GA: Gradient artifact
GGE: Genetic generalized epilepsy
GSWD: Generalized spike-wave discharges
HC: Healthy controls
hd-EEG: High density electroencephalography
ICA: Independent component analysis
ICC: Intracalcarine cortex
ImCoh: Imaginary part of coherency
MNI: Montreal Neurological Institute
MR(I): Magnetic resonance (imaging)
OBS: Optimal basis set
ROI: Region of interest
PALM: Permutation Analysis of Linear Models
PCC: Precuneus cortex
rsFC: Resting-state functional connectivity
sbFC: Seed-based functional connectivity
SUMA: Surface-based mapping, part of toolbox “Analysis of Functional NeuroImages
