# Supplementary material for: Comparison of Resting-State EEG Network Analyses With and Without Parallel MRI in Genetic Generalized Epilepsy
Source: Brain Topogr. 2023 Jun 24;36(5):750–65. doi: 10.1007/s10548-023-00977-6 (PMC10415462; doi:10.1007/s10548-023-00977-6)
Supplement: Supplementary file 1 — Supplementary file1 (DOCX 7609 KB) [file 10548_2023_977_MOESM1_ESM.docx]

## Appendix


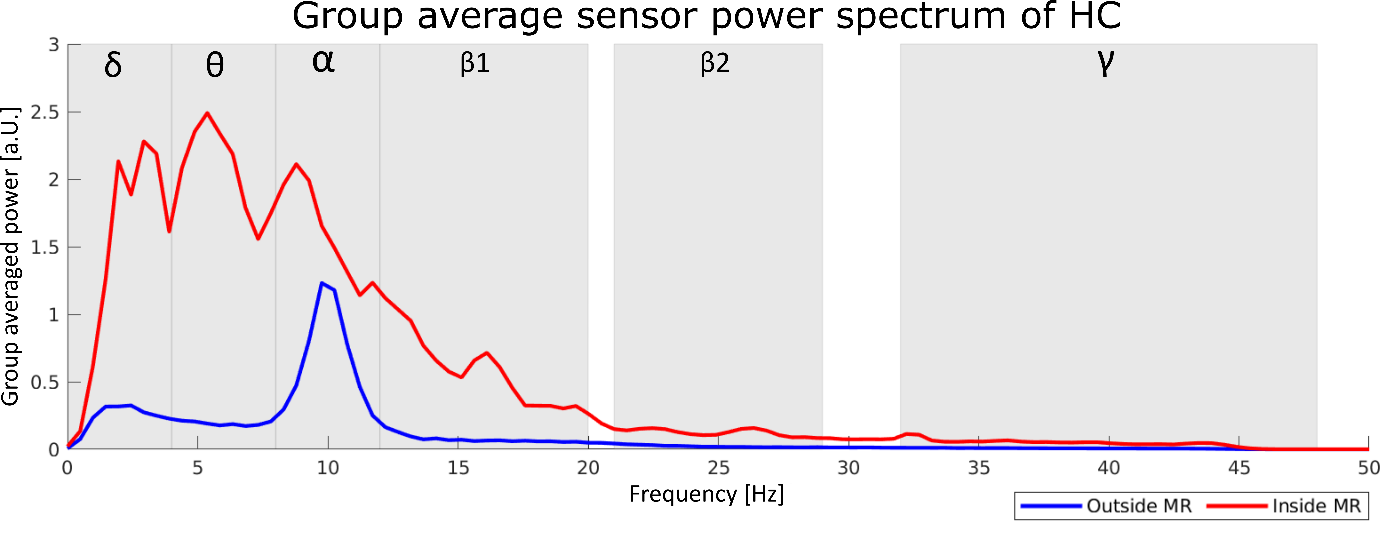


**Supplementary figure 1: Group-averaged sensor power spectrum for inside- and outside-MR scanner condition.**

Note the generally stronger power values in the inside condition compared within the outside condition, and the peaks in the inside-MR scanner condition in the frequency bands: delta (3 Hz), theta (5.5 Hz), alpha (8 Hz, 11.5 Hz) and beta1 (16 Hz).

**Supplementary table 1: 2-way ANOVA analysis of global power and functional connectivity values in each frequency**

*The effect of the measurement condition (main effect) was highly statistically significant for global power in all frequency bands. The interaction of the inside-outside factor on the patient-control group was only significant for global power in Beta1 (*$p$ *= 0.0447) and, at trend level, for Delta (*$p$ *= 0.0739). None of these interactions survived FDR correction. For global connectivity, the effect of the measurement condition was highly statistically significant in the delta and theta frequency bands after correcting for multiple comparison via FDR, and, at trend level, for Beta1 and Beta2. No statistically significant for the interaction of the inside-outside factor on the patient-control group was observed.*

|  | **Delta** | **Theta** | **Alpha** | **Beta1** | **Beta2** | **Gamma** |
| --- | --- | --- | --- | --- | --- | --- |
| **Global Power** |  |  |  |  |  |  |
| Main effect of inside/outside MR  condition |  |  |  |  |  |  |
| $p$  * $p$ < 0.1 , trend level  ** $p$ < 0.05 , significant | **0.00012 | **0.00015 | **0.00002 | **0.00006 | **0.00002 | **0.00012 |
| FDR corrected | **< 0.001 | **< 0.001 | **< 0.001 | **< 0.001 | **< 0.001 | **< 0.001 |
|  |  |  |  |  |  |  |
| Interaction of inside/outside MR  factor with group difference |  |  |  |  |  |  |
| $p$  * $p$ < 0.1 , trend level  ** $p$ < 0.05 , significant | *0.0739 | 0.111 | 0.136 | **0.0447 | 0.148 | 0.119 |
| FDR corrected | 0.148 | 0.148 | 0.148 | 0.148 | 0.148 | 0.148 |
|  |  |  |  |  |  |  |
| **Global functional connectivity** |  |  |  |  |  |  |
| Main effect of inside/outside MR  condition |  |  |  |  |  |  |
| $p$  * $p$ < 0.1 , trend level  ** $p$ < 0.05 , significant | **<0.0001 | **0.0002 | 0.6960 | *0.0655 | **0.0466 | 0.2340 |
| FDR corrected | **< 0.0001 | **< 0.001 | 0.696 | *0.09825 | *0.0932 | 0.2808 |
|  |  |  |  |  |  |  |
| Interaction of inside/outside  MR factor with group difference |  |  |  |  |  |  |
| $p$  * $p$ < 0.1 , trend level  ** $p$ < 0.05 , significant | 0.351 | 0.786 | 0.641 | 0.7249 | 0.798 | 07739 |
| FDR corrected | 0.798 | 0.798 | 0.798 | 0.798 | 0.798 | 0.798 |

**Supplementary table 2: Percentage differences of global power group mean between conditions for all frequencies**

|  | | Delta | Theta | Alpha | Beta1 | Beta2 | Gamma |
| --- | --- | --- | --- | --- | --- | --- | --- |
| Outside-MR scanner condition | HC | 2.1112 | 2.0046 | 2.1384 | 1.6262 | 1.3325 | 0.8164 |
|  | GGE | 2.3866 | 2.3929 | 2.5071 | 1.9374 | 1.5747 | 1.0298 |
|  | |  |  |  |  |  |  |
| Inside-MR  scanner condition | HC | 2.9626 | 2.9938 | 2.9201 | 2.5125 | 1.9875 | 1.4671 |
|  | GGE | 3.0470 | 3.1542 | 3.0965 | 2.5619 | 2.1130 | 1.5739 |
|  | |  |  |  |  |  |  |
| Group mean change between conditions | HC | 40% | 49% | 37% | 55% | 49% | 80% |
|  | GGE | 28% | 32% | 24% | 32% | 34% | 53% |

**Supplementary table 3: Percentage difference of global functional connectivity group mean between conditions for all frequencies**

|  | | Delta | Theta | Alpha | Beta1 | Beta2 | Gamma |
| --- | --- | --- | --- | --- | --- | --- | --- |
| Outside-MR scanner condition | HC | 0.0199 | 0.0233 | 0.0388 | 0.0268 | 0.0267 | 0.0277 |
|  | GGE | 0.0212 | 0.0277 | 0.0420 | 0.0288 | 0.0259 | 0.0254 |
|  | |  |  |  |  |  |  |
| Inside-MR scanner condition | HC | 0.0248 | 0.0276 | 0.0357 | 0.0286 | 0.0282 | 0.0275 |
|  | GGE | 0.0250 | 0.0339 | 0.0424 | 0.0325 | 0.0304 | 0.0278 |
|  | |  |  |  |  |  |  |
| Group mean change between conditions | HC | 25% | 18% | -8% | 7% | 6% | -1% |
|  | GGE | 18% | 22% | 1% | 13% | 17% | 10% |

*
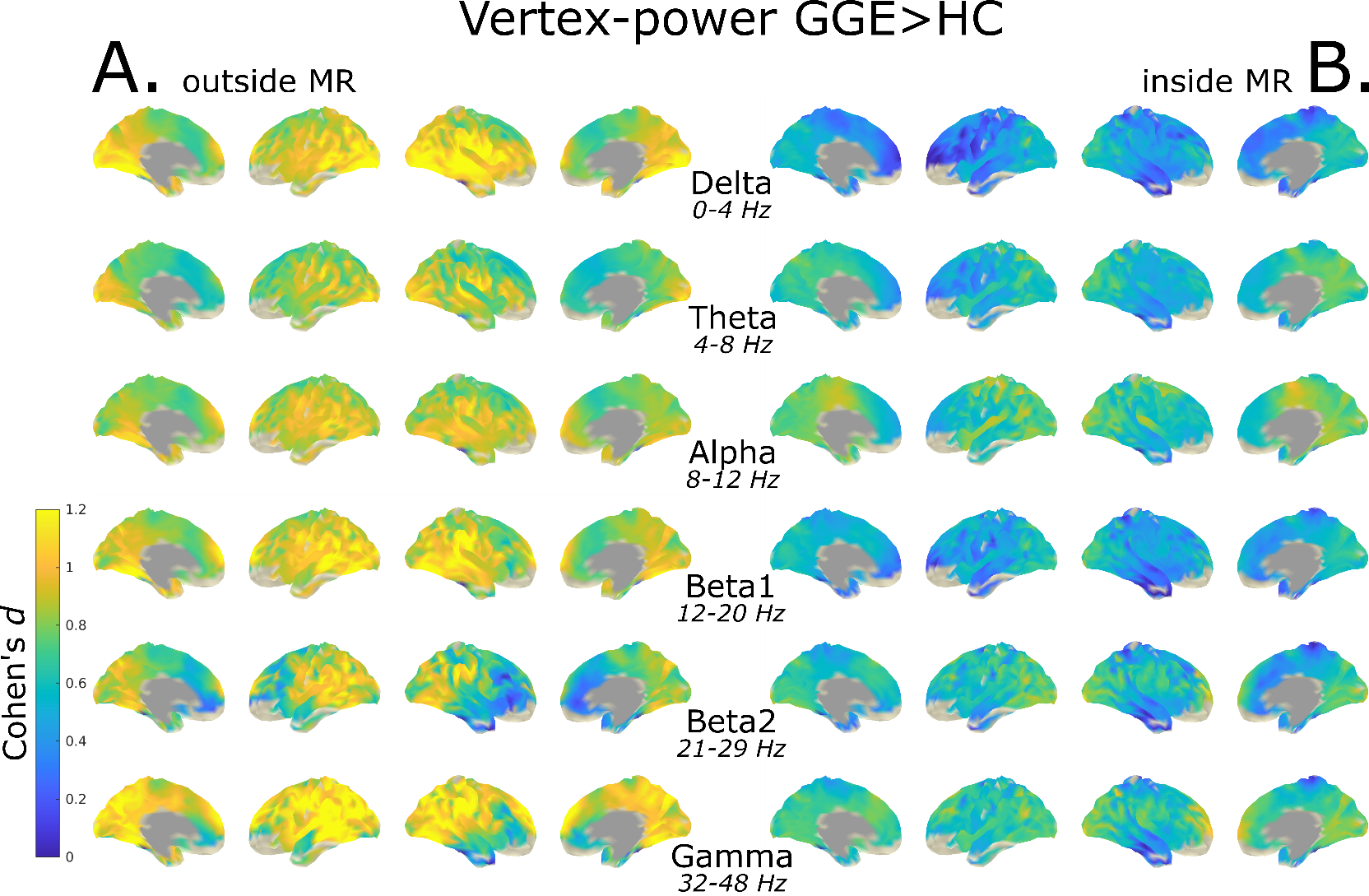
*

***Supplementary figure 2: Vertex-power group analysis results (GGE>HC) of inside- and outside-MR scanner conditions***

Standardized effect sizes (Cohen’s d) for group differences (GGE>HC) of vertex-based power for the outside- (A) and inside-MR condition (B). Note the effect size values $d$ in the inside scanner condition are smaller than in to the outside condition. They still show strong effect sizes (d > 0.8) in fewer but identical regions.

**Supplementary table 4: Spatial Pearson correlation of the cortical maps of group difference maps (**$\boldsymbol{-}\boldsymbol{log}_{\boldsymbol{10}}\boldsymbol{p}$**-value maps, GGE>HC) of power and ImCoh in each frequency band**

| ** $\boldsymbol{p}$_FDR_ < 0.001 | Power | ImCoh |
| --- | --- | --- |
| Delta | r = 0.8613 ** | 0.0978 ** |
| Theta | r = 0.8615 ** | 0.5476 ** |
| Alpha | r = 0.9127 ** | 0.1664 ** |
| Beta1 | r = 0.8916 ** | 0.2112 ** |
| Beta2 | r = 0.9070 ** | 0.2268 ** |
| Gamma | r = 0.9133 ** | 0.0831 ** |
